# Seroepidemiology of Saffold Cardiovirus Type 2

**DOI:** 10.3201/eid1708.101953

**Published:** 2011-08

**Authors:** Jochem Galama, Kjerstin Lanke, Jan Zoll, Merja Roivainen, Frank van Kuppeveld

**Affiliations:** Author affiliations: Radboud University Nijmegen Medical Center, Nijmegen, the Netherlands (J. Galama, K. Lanke, J. Zoll, F. van Kuppeveld);; Nijmegen Center for Molecular Life Sciences, Nijmegen (J. Galama, K. Lanke, J. Zoll, F. van Kuppeveld);; Nijmegen Institute for Infection, Inflammation and Immunity, Nijmegen (J. Galama, K. Lanke, J. Zoll, F. van Kuppeveld);; National Institute for Health and Welfare, Helsinki, Finland (M. Roivainen)

**Keywords:** viruses, Picornavirus, cardiovirus, Saffold virus, epidemiology, genotype, serotype, virus neutralization test, viruses, letter

**To the Editor:** Saffold virus (SAFV) is a new human virus belonging to the genus *Cardiovirus* of the family *Picornaviridae* (*1**–**6*). The virus has also been named human Theiler’s-like cardiovirus (*4*). To date, 8 SAFV genotypes are known, based on molecular variation in the P1 region, which codes for the viral capsid (*5*). SAFVs are detected in respiratory and fecal samples from infants and children <6 years of age. The virus is generally detected at low frequency (0.5%–3%) in the general population, but in Afghanistan and Pakistan higher incidences have been reported (10%–12%) (*5*). Detection is mainly based on molecular techniques because virus isolation is cumbersome. Other than gastroenteritis, no clear disease manifestations are known (*1**–**8*), although recently, researchers in Japan have suggested that for a select group of children, SAFV infection may cause pharyngitis and tonsillitis (*7*). In rodents, however, cardioviruses are serious pathogens that can cause myocarditis, meningoencephalitis, and pancreatitis.

Previously, we have shown that SAFV-3 infections are ubiquitous and are transmitted early in life (*6*). This conclusion was based on antibody testing by virus neutralization, a highly specific test that can discriminate serotypes within a single species, but formal proof for existence of serotypes was lacking. Extrapolated to other genotypes, the finding would indicate a high infection rate for SAFVs, which is at odds with the low detection frequency reported in most studies. Most SAFV-2 isolates, from our laboratory and others, grow poorly in cell culture, which hampers reading of neutralization. Recently, however, a SAFV-2 strain was isolated in Finland that grows well in HeLa cells and shows clear cytopathic effects (SAFV-2-FIN2008, GenBank accession no. FR682076; S. Blomgvist et al., unpub. data). This strain enabled us to set up a virus neutralization test similar to that described for SAFV-3 (*6*). For comparison, we used strain SAFV-3(NL2007) (GenBank accession no. FM207487), which was isolated in Nijmegen (*6*). The virus neutralization test was performed on HeLa cells with 100 TCID_50_ of virus per serum dilution. Virus–serum mixtures were incubated for 1 hour at 37°C and overnight at 7°C to stabilize virus–antibody complexes (*6*). Human serum samples submitted previously were tested by using 3-fold dilution steps and duplicate testing (*6*). The results are presented in the Table. A low seroprevalence was found for SAFV-2 and SAFV-3 at 9 months of age, which is at the nadir of immunoglobulin G levels in infants. At the age of 24 months, a high seroprevalence of antibodies was found in the Netherlands for SAFV-2 and -3, pointing to early acquisition of infection with both strains. In Finland, the seroprevalence for SAFV-2 and -3 was lower in young children, which suggests somewhat lower infection rate similar to what has been reported for enteroviruses (*9*). A high seroprevalence of 97%–100% was found in persons >4 years of age in Cameroon, Indonesia, and the Netherlands (Table). A high prevalence of SAFV-2 antibodies was recently also reported in blood donors from the United States (*8*). Thus, the seroepidemiology of SAFV-2 is similar to that of SAFV-3. As depicted in the last 3 columns of the Table, on several occasions serum samples independently neutralized either SAFV-2 or SAFV-3, which suggests that the viruses behave as different serotypes.

**Table:** SAFV-neutralizing antibodies in blood samples from humans of different ages and from different geographic regions*

Country	No. samples	Patient age†	Years collected	No. (%) samples				
				SAFV-2 pos‡	SAFV-3 pos‡	SAFV pos§	SAFV-2 pos + SAFV-3 neg	SAFV-2 neg + SAFV-3 pos
Netherlands	29	9 mo	2006–2007	15 (52)	4 (14)	15 (52)	11	0
Netherlands	26	24 mo	2006–2007	21 (81)	20 (77)	25 (96)	5	4
Netherlands	30	18–39 y	2004	30 (100)	29 (97)	30 (100)	1	0
Finland	30	2–2.5 y	1997–1998	10 (33)	21 (70)	24 (80)	3	14
Cameroon	29	5–15 y	1997	28 (97)	28 (97)	29 (100)	1	1
Indonesia	30	4–40 y	1997–1998	30 (100)	30 (100)	30 (100)	0	0

*SAFV, Saffold virus; pos, positive; neg, negative. †Samples were collected from patients at this age or within this age range. ‡Titer >15. §Cumulative positive for antibodies against SAFV-2 and/or SAFV-3.

In conclusion, SAFV-2 and SAFV-3 show an almost identical epidemiologic pattern with infection acquired early in life and with a high seroprevalence in different continents. The outcome is concordant with universal occurrence of infection by both genotypes. Because several times there was a clear discrepancy between antibody titers against one or the other of the 2 genotypes, we conclude that they behave as separate serotypes, although weak cross-reactivity (below the detection limit of 1:15) cannot be excluded. Accepting that SAFV genotypes correspond with existence of different serotypes, the infection rate in the first years of life must be quite high, similar to that for human parechoviruses and enteroviruses, which are found in 15%–18% of stool samples from children <5 years of age (*10*). The low SAFV detection rate remains thereby difficult to explain.

The outcome can be explained by a short duration of virus excretion in stool, which, however, is unlikely for an infection spreading by the fecal–oral route. Alternatively, it may be that the virus is unstable in stool and rapidly degrades, such that fecal samples are inadequate for diagnosis of the infection. Other specimens, however, such as respiratory samples, yielded also low numbers of positive findings (*4*). Remarkably, the study with a high prevalence of positive stool samples made use of primers selected in a conserved region of 2C helicase (*5*), whereas other studies used primers in the 5′ noncoding region (*3**,**4*). Hence, a difference in sensitivity between the different PCRs may be responsible for the discrepancy between seroepidemiology and the low diagnostic yield by PCR. This discrepancy, however, awaits further investigation.
